# Identification of Mutation Regions on *NF1* Responsible for High- and Low-Risk Development of Optic Pathway Glioma in Neurofibromatosis Type I

**DOI:** 10.3389/fgene.2018.00270

**Published:** 2018-07-24

**Authors:** Min Xu, Hui Xiong, Yanfang Han, Chijun Li, Shaozhen Mai, Zhongzhou Huang, Xuechen Ai, Zhixuan Guo, Fanqin Zeng, Qing Guo

**Affiliations:** ^1^Department of Dermatology, Sun Yat-sen Memorial Hospital, Sun Yat-sen University, Guangzhou, China; ^2^Department of Dermatology, The Eighth Affiliated Hospital, Sun Yat-sen University, Guangzhou, China

**Keywords:** neurofibromatosis type I, *NF1*, genotype, mutation, optic pathway glioma, phenotype

## Abstract

Neurofibromatosis type I is a rare neurocutaneous syndrome resulting from loss-of-function mutations of *NF1*. The present study sought to determine a correlation between mutation regions on *NF1* and the risk of developing optic pathway glioma (OPG) in patients with neurofibromatosis type I. A total of 215 patients with neurofibromatosis type I, from our clinic or previously reported literature, were included in the study after applying strict inclusion and exclusion criteria. Of these, 100 patients with OPG were classified into the OPG group and 115 patients without OPG (aged ≥ 10 years) were assigned to the Non-OPG group. Correlation between different mutation regions and risk of OPG was analyzed. The mutation clustering in the 5′ tertile of *NF1* was not significantly different between OPG and Non-OPG groups (*P* = 0.131). Interestingly, patients with mutations in the cysteine/serine-rich domain of *NF1* had a higher risk of developing OPG than patients with mutations in other regions [*P* = 0.019, adjusted odds ratio (OR) = 2.587, 95% confidence interval (CI) = 1.167–5.736], whereas those in the HEAT-like repeat region had a lower risk (*P* = 0.036, adjusted OR = 0.396, 95% CI = 0.166–0.942). This study confirms a new correlation between *NF1* genotype and OPG phenotype in patients with neurofibromatosis type I, and provides novel insights into molecular functions of neurofibromin.

## Introduction

Neurofibromatosis type I (MIM entry: 162200) is a rare disease with autosomal dominant inheritance that belongs to a neurocutaneous syndrome characterized by café-au-lait spots, skin freckling, neurofibroma, Lisch nodules, and optic pathway glioma (OPG) (Jett and Friedman, 2010). Its incidence and prevalence are 1/2500 and 1/4000, respectively (Pasmant et al., 2012). Neurofibromatosis type I results from loss-of-function mutations of the tumor suppressor *NF1* gene encoding neurofibromin (Trovó-Marqui and Tajara, 2006). Neurofibromin has several domains and special structures; its main role is to act as a negative regulator in the Ras pathway (Ratner and Miller, 2015). Although the *NF1* sequence has been known for nearly 30 years, the correlation between genotype and phenotype in neurofibromatosis type I remains poorly understood. This is largely due to low incidence, the considerable length of *NF1*, diffused distribution of mutations, and the age-dependent variability of manifestations (Wallace et al., 1990). So far, the few published genotype–phenotype correlations have indicated a severe phenotype in patients with whole-gene deletion and the absence of cutaneous neurofibroma in patients with a 3-bp inframe deletion (c.2970–2972 delAAT) or missense mutation (p.Arg1809Cys) (Cnossen et al., 1997; Upadhyaya et al., 2007; Mautner et al., 2010; Pinna et al., 2014).

Recently, two studies suggested that patients with mutations in the 5′ tertile (exon 1–21) of *NF1* had a higher risk of developing OPG (Sharif et al., 2011; Bolcekova et al., 2013). OPG is a type of pilocytic astrocytoma located in the cerebral optic pathway, which can impair patients’ vision and visual field (Listernick et al., 2007). However, approximately 50–75% of patients present no symptoms at the time OPG is diagnosed (Sylvester et al., 2006), making it important to evaluate the risk of OPG in asymptomatic patients. Notably, a subsequent study based on stricter inclusion criteria, revealed that mutation clustering in the 5′ tertile was common to both the OPG and control groups (Hutter et al., 2016). These conflicting results show that further studies are required to establish a firm correlation between *NF1* genotype and OPG.

Previous studies were limited by either small samples or poor inclusion criteria, both of which could lead to biased results. In the present effort, we used strict inclusion criteria, which we applied also to patients from previous studies, to obtain a sufficiently large cohort. Furthermore, the region comprising the 5′ tertile is fairly vast, which might have affected the accuracy of previous results. Instead, here we applied an established practice common to the study of hereditary diseases (Hateboer et al., 2000; Tartaglia et al., 2002; Aartsma-Rus et al., 2006; Mori-Yoshimura et al., 2012), and focused specifically on correlations between mutations in regions encoding protein domains or other regions of the *NF1* gene and the OPG phenotype in patients with neurofibromatosis type I.

## Materials and Methods

### Literature Search

A thorough search of English-language literature for relevant papers which focused on the genotype–phenotype correlation or described clinical manifestations and gene mutations in patients with neurofibromatosis type I was performed. Databases such as PubMed and Google Scholar, as well as university library resources were searched. Search terms included “(OPG OR optic pathway glioma OR optic glioma) AND (nf1 OR neurofibromatosis) AND (genotype OR mutation),” “(glioma OR optic nerve tumor OR optic nerve glioma) AND (nf1 OR neurofibromatosis) AND (genotype OR mutation),” “(nf1 OR neurofibromatosis) AND (OPG OR optic pathway glioma OR optic glioma),” and “(nf1 OR neurofibromatosis) AND (glioma OR optic nerve tumor OR optic nerve glioma).” All related papers were read through to evaluate whether the reported patients were compatible with the present study’s inclusion criteria.

### Study Subjects

Strict inclusion criteria included: (1) conformity to NIH diagnostic criteria for neurofibromatosis type I (Conference, 1988); (2) brain radiology (computed tomography or magnetic resonance imaging) to diagnose or exclude OPG; (3) *NF1* gene test to determine pathogenic germline mutations; (4) age of patients without OPG of 10 years or more (rare patients with neurofibromatosis type I could develop OPG after that point) (Nicolin et al., 2009; Hutter et al., 2016).

Exclusion criteria included: (1) inconclusive radiological diagnosis of OPG; (2) wrong assessment of gene mutations, due to cDNA changes not conforming to predicted amino acid alterations or the original cDNA base in the reported position not conforming to the corresponding base in the reference sequence; (3) existence of two variants of *NF1* in a patient for which the pathogenic one could not be determined according to present evidence; (4) patients in a study which only focused on a certain point mutation, because these patients were selected artificially.

After selection following the above inclusion and exclusion criteria, five patients from our clinic and 210 patients described in the literature were included in this study. Of these, 100 patients were classified in the OPG group and 115 in the Non-OPG group. The five patients from our clinic were scanned to obtain brain magnetic resonance imaging and 2 mL of blood was collected for *NF1* gene testing using next-generation sequencing (NGS) in an Illumina HiSeq 2000 system (Illumina Corporation, San Diego, CA, United States), and small mutations were verified using Sanger sequencing among probands’ family members. Multiplex ligation-dependent probe amplification (MLPA) was used to detect the large rearrangement.

In summary, small mutations and large rearrangements of *NF1* were detected in 208 and 7 patients, respectively. Among the 208 patients with small mutations, the most common methods for ascertaining mutations were Sanger sequencing (114, 54.81%) and NGS (55, 26.44%), whereas the sequencing methods of the remaining 39 patients (18.75%) were not reported in the literatures. Among the seven patients with large rearrangements, the most common methods for detecting mutations were MLPA (6, 85.71%), followed by single nucleotide polymorphism array (1, 14.29%).

### Mutation Analysis

Each *NF1* gene mutation was verified and analyzed in Mutalyzer^1^ and all mutation information (c.DNA position and base changes) was transformed to novel nomenclature using transcript number NG_009018.1 (NF1_v002; Anastasaki et al., 2017). Furthermore, mutations were also analyzed using MutationTaster^2^, which predicted amino acid alterations. Exons were divided into 5′ tertile (exon 1–21), middle tertile (exon 22–38), and 3′ tertile (exon 39–57) for mutation clustering analysis (Sharif et al., 2011). The splice site mutation was classified to the consensus exon for statistical analysis. Additionally, to detect a correlation between *NF1* genotype and OPG phenotype, several gene regions were analyzed. These included the cysteine/serine-rich domain (CSRD, residues 543–909), tubulin-binding domain (TBD, residues 1095–1197), GTPase-activating protein-related domain (GRD, residues 1198–1530), Sec14-like domain (Sec14, residues 1560–1705), pleckstrin homology-like domain (PH, residues 1716–1816), HEAT-like repeat regions (HLR, residues 1825–2428), C-terminal domain (CTD, residues 2260–2817), nuclear localization signal region (NLS, residues 2534–2550), and syndecan-binding region (SBR, residues 2619–2719) (Vandenbroucke et al., 2004; Bonneau et al., 2009; Luo et al., 2014). The bipartite module with phospholipid binding activity consisted of Sec14 and PH; therefore, the area of Sec14-PH combined the regions of Sec14 and PH (D’Angelo et al., 2006). Patients with whole-gene deletions were not included in the analysis of mutation clustering in different tertiles or all gene regions.

### Statistical Analysis

This study was designed as a case control study. Data were analyzed using SPSS version 24.0 (IBM Corporation, Chicago, IL, United States) and GraphPad PRISM version 7.01 (GraphPad Software, San Diego, CA, United States). Constituent ratio and sector diagram were used to describe the distribution of mutation types. The number of patients in each group is presented in bar graphs. Contingency tables and Chi-square test were used for categorical variables. When any expected value on contingency tables was below five, results were corrected for continuity. When any expected value on contingency tables was below one or the total sample number was less than 40, Fisher’s exact test was used. Logistic regression was used to evaluate the risk of developing OPG due to mutations in each region, and crude odds ratios (ORs) were reported with 95% confidence intervals (CIs). Mutations in regions with significantly high or low risk of developing OPG were analyzed using multiple logistic regression, and adjusted ORs were also reported with 95% CI. *P*-values < 0.05 were considered statistically significant.

## Results

### Distribution of Mutation Types and Mutation-Containing Regions in Patients

**FIGURE 1:**
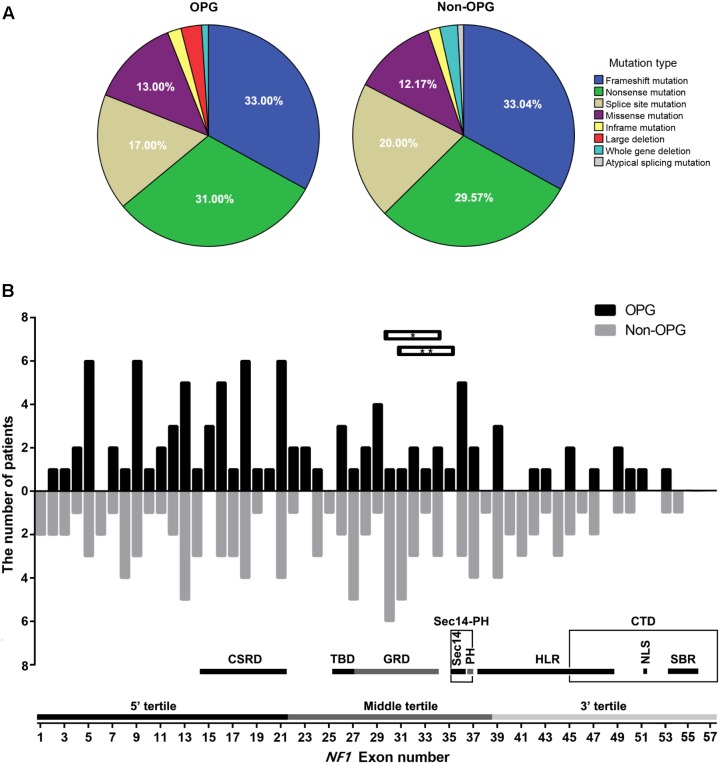
Distribution of mutation types and mutation-containing regions on the *NF1* gene in patients. **(A)** Percentage of different mutation types in the OPG and Non-OPG groups. **(B)** Bar graph showing the number of patients whose mutations were located within different exons in the OPG and Non-OPG groups. Splice site mutation was included in the consensus exon. Whole-gene deletion is not shown in this figure. Horizontal bar with^∗^ indicates one OPG patient with a large deletion of exons 30–34. Horizontal bar with^∗∗^ indicates one OPG patient with a large deletion of exons 31–35. A schematic diagram showing the number of exons contained in different regions or tertiles is presented. CSRD, cysteine/serine-rich domain; TBD, tubulin-binding domain; GRD, GTPase-activating protein-related domain; Sec14, Sec14-like domain; PH, pleckstrin homology-like domain; Sec14-PH, module combining Sec14 and PH; HLR, HEAT-like repeat regions; CTD, C-terminal domain; NLS, nuclear localization signal region; SBR, syndecan-binding region.

The number of patients with frameshift mutation, nonsense mutation, splice site mutation, missense mutation, inframe mutation, large deletion, whole-gene deletion, and atypical splicing mutation was 33 (33%), 31 (31%), 17 (17%), 13 (13%), 2 (2%), 3 (3%), 1 (1%), and 0, respectively, in the OPG group and 38 (33.04%), 34 (29.57%), 23 (20%), 14 (12.17%), 2 (1.74%), 0, 3 (2.61%), and 1 (0.87%), respectively, in the Non-OPG group. The distribution of mutation types did not differ significantly between the two groups (*P* = 0.696, **Figure 1A**). The distribution of mutation-containing regions for the two groups is shown in **Figure 1B**, and details are presented in Supplementary Table S1.

### Mutation Clustering in the 5′ Tertile Occurs in Both OPG and Non-OPG Groups

**FIGURE 2:**
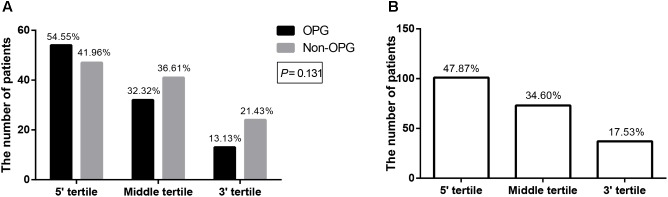
Bar graph depicting the number of patients with mutations located in different tertiles of *NF1*. The number of patients with mutations located in different tertiles of *NF1* in the OPG group versus Non-OPG group **(A)** and total patients **(B)**. The number above each bar represents the percentage in the relevant group.

Having assembled a large sample using strict inclusion criteria, we verified whether mutation clustering conformed to that in previous studies by comparing the number of patients from both groups, whose mutations were located in different tertiles (Sharif et al., 2011; Bolcekova et al., 2013; Hutter et al., 2016). Mutation clustering in the 5′ tertile was observed in both the OPG group and Non-OPG group, but the difference between them was not statistically significant (*P* = 0.131, **Figure 2A**). The total number of patients from both groups, presenting mutations located in the 5′ tertile, middle tertile, or 3′ tertile was 101 (47.87%, 95% CI = 41.07–54.66%), 73 (34.60%, 95% CI = 28.13–41.67%), and 37 (17.53%, 95% CI = 12.36–22.71%), respectively (**Figure 2B**). Thus, mutation clustering in the 5′ tertile was characteristic of all patients and not only of those in the OPG group.

### Patients With Mutations in the CSRD Are at Higher Risk of Developing OPG, Those in the HLR Are at Lower Risk

A protein domain is defined as the functional region of a protein; accordingly, mutations affecting protein domain regions can directly disrupt the function of a protein (Welti et al., 2011). We analyzed all common domain regions of *NF1* to evaluate the OPG risk of mutations in these regions (**Table 1**). Patients with mutations in the CSRD correlated with a higher risk of developing OPG than patients with mutations in other regions (*P* = 0.019, adjusted OR = 2.587, 95% CI = 1.167–5.736). Patients with mutations in Sec14-PH displayed a tendency toward a higher risk of developing OPG than patients with mutations in other regions; however, this trend was not statistically significant (*P* = 0.057, adjusted OR = 3.712, 95% CI = 0.961–14.332). Instead, patients with mutations in the HLR had a lower risk of developing OPG than patients with mutations in other regions (*P* = 0.036, adjusted OR = 0.396, 95% CI = 0.166–0.942). The remaining gene regions did not reveal any significantly different mutation distributions between the two groups. Thus, the risk of developing OPG in patients with neurofibromatosis type I correlated with mutations in the CSRD and HLR.

**Table 1:** Mutations in different *NF1* gene regions and their risk of developing OPG.

Regions	OPG n (%) *N* = 99	Non-OPG, n (%) *N* = 112	^#^*P*-value	^##^Crude OR (95% CI)	^##^*P*-value	^###^Adjusted OR (95% CI)	^###^*P-*value
CSRD	23 (23.23)	11 (9.82)	0.008	2.779 (1.277–6.048)	0.010	2.587 (1.167–5.736)	0.019
TBD	3 (3.03)	8 (7.14)	0.180	0.406 (0.105–1.576)	0.193		
GRD	15 (15.15)	22 (19.64)	0.392	0.731 (0.355–1.502)	0.393		
Sec14-PH	9 (9.09)	3 (2.68)	0.045	3.633 (0.955–13.822)	0.058	3.712 (0.961–14.332)	0.057
HLR	8 (8.08)	25 (22.32)	0.004	0.306 (0.131–0.715)	0.006	0.396 (0.166–0.942)	0.036
CTD	8 (8.08)	9 (8.04)	0.990	1.006 (0.373–2.716)	0.990		
NLS	0	0	–	–	–		
SBR	1 (1.01)	2 (1.79)	>0.999^∗^	0.561 (0.05–6.285)	0.639		
Others	37 (37.37)	40 (35.71)	0.803	0.931 (0.531–1.632)	0.803		


*Differences between the frequencies of mutation in different gene regions of *NF1* in the OPG and Non-OPG groups. Patients with a whole-gene deletion were not included. Some mutations’ location may overlap different regions as indicated in **Figure 1B**. ^∗^*P-*value used continuity correction, ^#^*P* used chi-square test, ^##^*P* used binary logistic regression, ^###^*P* used multinomial logistic regression. OPG, optic pathway glioma; OR, odds ratio; CI, confidence interval; CSRD, cysteine/serine-rich domain; TBS, tubulin-binding domain; GRD, GTPase-activating protein-related domain; Sec14, Sec14-like domain; PH, pleckstrin homology-like domain; Sec14-PH, Sec14-PH module; HLR, HEAT-like repeat region; CTD, C-terminal domain; NLS, nuclear localization signal domain; SBR, syndecan-binding region.*

### Distribution of Mutation Types in the CSRD and HLR

As different mutation types exert a different effect on protein structure and function (Richards et al., 2015), we analyzed the distribution of mutation types in gene regions where disruptions would lead to high or low risk of developing OPG (**Table 2**). In spite of the higher frequency of nonsense mutations within the CSRD in the OPG group and higher frequency of splicing site mutations within the HLR in the Non-OPG group, no significant differences were detected (*P* > 0.05). Thus, there was not enough evidence to suggest that a certain mutation type contributed in a predominant way to the high or low risks of developing OPG in these two gene regions.

**Table 2:** Distribution of mutation types in the CSRD and HLR between the OPG and Non-OPG groups.

Mutation type	CSRD		HLR	
		
	OPG, n (%) *N* = 23	Non-OPG, n (%) *N* = 11	OPG, n (%) *N* = 8	Non-OPG, n (%) *N* = 25
Frameshift mutation	10 (43.48)	6 (54.55)	2 (25.00)	7 (28.00)
Nonsense mutation	5 (21.74)	1 (9.09)	4 (50.00)	10 (40.00)
Splice site mutation	3 (13.04)	2 (18.18)	1 (12.50)	7 (28.00)
Missense mutation	4 (17.39)	2 (18.18)	0	1 (4.00)
Large deletion	1 (4.35)	0	0	0
Inframe mutation	0	0	1 (12.50)	0
Atypical splicing mutation	0	0	0	0
*P* value^∗^	0.934		0.517	


*^∗^*P-*values used Fisher exact test. CSRD, cysteine/serine-rich domain; HLR, HEAT-like repeat region; OPG, optic pathway glioma.*

## Discussion

The impact of a specific gene mutation on the corresponding protein has been shown to vary with the location of the mutation (Hateboer et al., 2000; Tartaglia et al., 2002; Aartsma-Rus et al., 2006; Mori-Yoshimura et al., 2012). In this study, a large cohort of patients assembled using strict inclusion and exclusion criteria was analyzed to evaluate the correlation between mutation-containing regions and the risk of developing OPG. Results indicated mutation clustering in the 5′ tertile of *NF1* existed in both the OPG group and Non-OPG group; however, our study newly reported that patients with mutations in the CSRD and HLR of *NF1* exhibited a higher and lower risk, respectively, of developing OPG than patients with mutations in other regions, irrespective of mutation type.

Optic pathway glioma is the most common central nervous system tumor in patients with neurofibromatosis type I. Several studies have attempted to reveal a correlation between genotype and phenotype in neurofibromatosis patients with OPG (Sharif et al., 2011; Bolcekova et al., 2013; Hutter et al., 2016); however, results have been mostly inconclusive, probably due to the small number of participants and varying inclusion criteria. Furthermore, inclusion criteria may have not been sufficiently rigorous in these studies, and included lack of a radiology-based diagnosis of OPG or the presence of Non-OPG patients younger than 10 years. These, may have resulted in wrong classification of patients in the OPG or Non-OPG groups, thus causing bias in the results (Sharif et al., 2011; Bolcekova et al., 2013). Here, we included a larger sample size using strict inclusion and exclusion criteria. Our results demonstrated no correlation between mutations clustering in the 5′ tertile of *NF1* and the risk of OPG, thus confirming the study by Hutter et al. (2016).

The region comprising the 5′ tertile is fairly vast and may affect the accuracy of the results. To obtain more precise results, we analyzed correlations between mutations either in regions encoding protein domains or other regions of *NF1* and the OPG phenotype. CSRD is a unique domain located at the N-terminus of neurofibromin and its function is not entirely clear. CSRD, with its three cysteine pairs (residues 622/632, 673/680, and 714/721), could be phosphorylated by cAMP-dependent protein kinase A (PKA) and protein kinase C (PKC) (Marchuk et al., 1991; Fahsold et al., 2000; Mangoura et al., 2006). PKC-dependent phosphorylation of the CSRD could enhance the biological effect of neurofibromin through interactions with the actin cytoskeleton and the allosteric effect on its Ras-GAP domain (GRD), which could reduce Ras activity and suppress tumorigenesis (Mangoura et al., 2006). Mutations affecting the CSRD might limit the biological effect of neurofibromin, possibly explaining why mutations in the CSRD were associated with a higher risk of developing OPG. Phosphorylation of the CSRD regulates GRD activity, but mutations in the latter did not increase the risk of developing OPG to the same extent as did mutations in the former. This observation is hard to reconcile at present, but suggests that heterozygous mutations in the CSRD might be more harmful than heterozygous mutations in the GRD in the tumorigenesis of OPG, which should be investigated further.

Sec14-PH was reported as a bipartite module of neurofibromin responsible for binding to cellular phospholipids and possibly the membrane localization of neurofibromin (Welti et al., 2007, 2008). Our results showed a tendency toward a higher risk of developing OPG in patients with mutations in the Sec14-PH; however, this trend was not statistically significant and might require verification in a larger cohort. Besides regions whose disruption conferred a higher risk of OPG, mutations in the HLR appeared to associate with a lower risk of developing OPG compared with mutations in other regions. HLR is a newly discovered structure, responsible for interactions between proteins and different molecules in a variety of proteins (Kajava et al., 2004; Jeong et al., 2013; Hadpech et al., 2017). The structure of the HLR is highly similar to that of HEAT repeats with repetitive arrays of short amphiphilic α-helices, and it exists in a wide variety of eukaryotic proteins, allowing them to adapt to both hydrophilic and hydrophobic environments (Yoshimura and Hirano, 2016). Until now, little has been known about the molecular function of the HLR in neurofibromin. Our results indicate that, contrary to the CSRD, mutations in this region were associated with a lower risk of OPG than mutations in other regions. This finding suggests that the HLR might perform a negative feedback function on neurofibromin in astrocytes. We believe that this hypothesis warrants further consideration in future experiments. It should be noted that the risk associated with mutations in the CSRD and HLR of *NF1* can also be observed in participants examined in previous studies focusing on correlation between mutations in the 5′ tertile and OPG phenotype (Supplementary Table S2) (Bolcekova et al., 2013; Hutter et al., 2016).

Although different mutation types exert different effects on protein structure and function (Richards et al., 2015), we failed to observe any significant correlation between mutation type and OPG phenotype in mutations located in the CSRD or HLR. However, any tendency toward a correlation observed for some mutation types, requires verification with a larger sample, because subdivision per mutation type created groups of too small a size.

In the present study, we successfully decreased the bias resulting from diagnosis by tightening the inclusion and exclusion criteria. Nevertheless, some level of bias resulted from the different procedures and techniques used to quantify the pathogenic mutation. Even though more than half the patients were examined using Sanger sequencing, often regarded as gold standard for molecular diagnostics (Rehm, 2013), possible bias resulting from the different methods and procedures should be kept in mind. The other limitations of this study include: (1) no consideration of racial difference as a possible variable for broadening sample size; (2) lack of a clear correlation between the risk of developing OPG and other mutations not located in the CSRD or HLR, but which also affected the function of these regions.

## Conclusion

The present study newly revealed that neurofibromatosis type I patients with mutations located in the CSRD and HLR of the *NF1* gene had a higher and lower risk than patients with mutations in other regions, respectively, of developing OPG. Thus in clinic, we should pay more attention to patients with mutations in CSRD to evaluate the development of OPG. This study suggests that a precise strategy is important in the analysis of genotype–phenotype correlations of *NF1* and provides novel insights into the molecular functions of neurofibromin.

## Ethics Statement

This study was carried out in accordance with recommendations of ‘the Ethics Committee of Sun Yat-sen Memorial Hospital’ with written informed consent from all our subjects. All our patients gave written informed consent in accordance with the Declaration of Helsinki. The protocol was approved by the ‘the Ethics Committee of Sun Yat-sen Memorial Hospital’.

## Author Contributions

MX designed the study and drafted the manuscript. MX, HX, and YH searched for previously reported cases and analyzed the data. CL, SM, ZH, XA, and ZG verified the genetic data. FZ and QG supervised the study and assisted in revising the manuscript.

## Conflict of Interest Statement

The authors declare that the research was conducted in the absence of any commercial or financial relationships that could be construed as a potential conflict of interest.

## Footnotes

1 https://mutalyzer.nl/

2 http://www.mutationtaster.org/
