# Supplementary material for: Identification of Mutation Regions on NF1 Responsible for High- and Low-Risk Development of Optic Pathway Glioma in Neurofibromatosis Type I
Source: Front Genet. 2018 Jul 24;9:270. doi: 10.3389/fgene.2018.00270 (PMC6066643; doi:10.3389/fgene.2018.00270)
Supplement: Supplementary file 2 [file Table_2.PDF]

Table S2. Mutations in the CSRD and HLR between OPG and Non-OPG groups in previous studies by Bolcekova et al. and Hutter et al

Domain	OPG		Non-OPG			Total		
	Bolcekova et al, n(%) N=24	Hutter et al, n(%) N=37	Bolcekova et al, n(%) N=24	*Bolcekova et al, n(%) N=14	Hutter et al, n(%) N=33	Bolcekova et al, n(%) N=48	*Bolcekova et al, n(%) N=38	Hutter et al, n(%) N=70
CSRD	6 (25.00)	5 (13.51)	3 (12.50)	1 (7.14)	2 (6.06)	9 (18.75)	7 (18.42)	7 (10.00)
HLR	2 (8.33)	3 (8.11)	4 (16.67)	2 (14.29)	9 (27.27)	6 (12.50)	4 (10.53)	12 (17.14)

The number of patients in (*Bolcekova et al) was counted after the exclusion of patients aged less than 10 years in the Non-OPG group. CSRD: cysteine serine rich domain; Sec14-PH: Sec14-PH module; HLR: HEAT-like repeat region; OPG: optic pathway glioma
